# Identifying Factors Associated with Barriers in the Number of Antenatal Care Service Visits among Pregnant Women in Rural Parts of Ethiopia

**DOI:** 10.1155/2021/7146452

**Published:** 2021-10-25

**Authors:** Sali Suleman Hassen, Belete Mulatu Teshale, Lema Abate Adulo

**Affiliations:** ^1^Department of Statistics, College of Natural and Computational Science, Mizan-Tepi University, Tepi, Ethiopia; ^2^Department of Statistics, College of Natural and Computational Science, Mizan-Tepi University, Tepi, Ethiopia

## Abstract

**Background:**

Antenatal care visit is the service given to pregnant women to have a safe pregnancy and a healthy baby. The main objective of this study was to identify potential factors for the barriers in the number of antenatal care visits.

**Methods:**

Data for this study was taken from the 2016 Ethiopian demographic health survey. All childbearing women from rural parts of Ethiopia were considered in this study, and the count regression model was used to explore the major risk factors for the barriers in the number of antenatal care service visits.

**Results:**

Nearly 42% of pregnant mothers did not visit antenatal care services, and only 1% of the mothers attended antenatal care service visits eight times and above. From hurdle Poisson regression model results, women having previous pregnancy complication (AOR = 1.16; *P* ≤ 0.001); husbands with primary education (AOR = 1.02; *P*=0.004), secondary education (AOR = 1.117; *P* ≤ 0.0001), and higher education (AOR = 1.191; *P* ≤ 0.001); middle wealth index (AOR = 1.08; *P*=0.006); richer wealth index (AOR = 1.10; *P* ≤ 0.001); maternal age 35–49 (AOR = 0.690; *P* ≤ 0.001); being exposed to media access (AOR = 1.745; *P*=0.019); having distance problem (AOR = 0.75; *P*=0.013); planned pregnancy (AOR = 1.42; *P*=0.002); and mothers with primary education (AOR = 1.85; *P* ≤ 0.001) and secondary (AOR = 2.387; *P* ≤ 0.001) were statistically associated with barriers in the number of ANC service visits.

**Conclusion:**

As indicated in the findings, there is underutilization of the antenatal care service visits regarding rural women in Ethiopia. Having a low education level, no media access, distance problem from the health facility, and not planned pregnancy decrease the rate of antenatal care service visits. To fill this discrepancy, the concerned bodies including government and nongovernmental organizations should work on the identified factors in the rural parts of the country to save children and mothers.

## 1. Introduction

Antenatal care (prenatal care) is the care given to expectant mothers and adolescent girls by experienced healthcare personnel to ensure the greatest possible health for both mother and baby throughout pregnancy. It is prenatal care for pregnant women that involves education, counseling, screening, and treatment to monitor and promote the mothers' and fetus' well-being [1, 2]. Good antenatal care visits also allow pregnant women to speak with their healthcare practitioner, increasing the likelihood that they will use a trained birth attendant [2].

Even though antenatal care has such appealing benefits and tactics, 303,000 women have died globally as a result of pregnancy and childbirth-related issues in recent years, with 99 percent of maternal deaths occurring in developing nations [3, 4]. Ethiopia is one of the developing countries with the highest maternal death and morbidity rates. Attending the recommended number of antenatal care appointments may help to reduce maternal morbidity and mortality by detecting pregnancy-related issues and increasing the number of deliveries made at a health facility [5].

To reduce maternal morbidity and mortality, the 2016 World Health Organization ANC model states that a minimum of eight contacts is recommended for a positive pregnancy experience [1].

In Ethiopia, prenatal care utilization is low in comparison to WHO recommendations, and there is variation in ANC service utilization from region to region within the country [6]. In an Ethiopian study, it was discovered that 2,598 (36.6%) of women had used at least four ANC visits, and that place of residence, region, mothers education, household wealth index, desire for pregnancy, frequency of reading newspaper, frequency of listening to the radio, and frequency of watching television were all significantly associated with the use of ANC [7, 8].

According to a study conducted in Vietnam, mothers residing in rural regions were less likely to follow national recommendations for ANC service visits [9]. Furthermore, according to a study conducted in Ethiopia, just 23.3 percent of rural inhabitants completed four or more ANC visits, while 71.6 percent of urban residents completed the necessary four or more ANC visits. In comparison to urban women, rural women were 0.35 times less likely to complete four or more ANC visits (OR 0.35, CI: 0.22, 0.53) [10]. The study that took place in Northwest Ethiopia indicated that urban mothers were 9.54 times (AOR: 9.54; CI: 5.99, 15.17) more likely to have skilled delivery services in health institutions compared to their rural counterparts [11]. According to a study conducted in rural Ethiopia, rural Ethiopians had less access to modern ANC consultations during pregnancy than the national average. Lack of knowledge, lack of education, hard workload, poverty, and a scarcity of health positions were all associated with not attending ANC visits [12, 13]. To this end, this study assessed the factors associated with the barriers in the number of antenatal care service visits using the 2016 Ethiopian Demographic Health Survey data set and count regression models.

## 2. Methods

### 2.1. Study Design and Data Set

By eliminating mothers from metropolitan areas, this study was done in rural areas of nine regions and one city administration. 16,650 households were interviewed for a personal interview, and 16,583 suitable women were found from the interviewed households. Only 5,667 mothers from rural areas were examined to work out elements that generate barriers in the frequency of antenatal care visits out of a total of 15,683 children-bearing mothers interviewed.

The comprehensive report of the EDHS 2016, which was implemented by the Central Statistical Agency (CSA) at the request of the Federal Ministry of Health (FMOH) in 2016 and was the fourth inclusive survey, contained thorough reports on data management. The data was collected between January 18 and June 27, 2016, and is available online at https://www.dhsprogram.com/data/dataset_admin/login_main.cfm from the EDHS database.

### 2.2. Study Variables

The number of antenatal care visits attended by pregnant women from early pregnancy to 9 months of pregnancy was the dependent variable in this study. Thus, based on literature reviews and theoretical reasoning, this study attempted to cover socioeconomic, demographic, fertility, and ANC service-related characteristics that were thought to be potential predictors for differences in the number of prenatal care service visits. This study's response variable is *Y*_*i*_, which represents the number of prenatal care visits per pregnant woman in Ethiopia. As a result, *Y*_*i*_ takes the number of pregnant women attending ANC visits (*i*=0,1,2,…, 8) times.

Sociodemographic characteristics (maternal age, region, husband educational, mother education, wealth index, and media access), fertility-related characteristics (pregnancy complication), and ANC service-related characteristics (distance from a healthcare facility, plan of pregnancy, and peer influence) were expected to be the most determinants against attending antenatal care follow-up during this study.

### 2.3. Statistical Data Analysis

The SPSS version 20 and R-version 3.6.2 statistical tools were used to clean, code, and analyze the data taken from the EDHS 2016 for this study. The descriptive analysis employing frequency and percentage, as well as the Hurdle Poisson regression model from inferential statistics, were used to identify the risk factors for barriers within the number of antenatal care services. The predictor variables that were significant at the 25% (*P* value 0.25) level of significance in the univariable analysis were included in the multiple hurdle Poisson regression analysis. The calculated odds ratios and 95 percent confidence intervals in multivariable analysis with a *P* value less than 0.05 indicate that the variables are statistically significant and can be used to interpret the results.

### 2.4. Inclusion and Exclusion Criteria

The eligibility criteria were being an Ethiopian national, aged 15–49 years, had given birth or being pregnant during the year before the interview, and living in rural parts of Ethiopia during the time of pregnancy. This study excluded the mothers with any mental problem, under-15 aged girls, those women who suffer infertility, and those mothers who did not consent to participate.

### 2.5. Count Regression Models

For count data, the Poisson distribution is the most common probability distribution. For events that occur at random in time and/or space, the Poisson probability model is appropriate. Because the response variable (number of ANC visits) is a nonnegative integer, most contemporary thinking in the field suggests starting with a Poisson regression model. The likelihood of a pregnant woman visiting a prenatal care provider during her nine months of pregnancy (where is a nonnegative integer) is provided by

(1) $$\begin{array}{c} P\left(Y=y_{i}\right)=\frac{e^{\mu }\mu^{y}}{y!}, \end{array}$$

where *P*(*Y*=*y*_*i*_) in equation (1) is the probability of a pregnant mother entity *i* having antenatal care service visits in nine (9) months of pregnancy period and *μ* is the Poisson parameter for pregnant mothers, which is equal to the expected number of antenatal care service visits *E*(*yi*).

(2) $$\begin{array}{c} \mu =E\left(yi\right)=\exp\left(x_{i}^{T}\beta \right), \end{array}$$

where *X*_*i*_^*T*^=(1, *x*_*i*1_, *x*_*i*2_,…, *x*_*ip*_) is a vector of explanatory variables and *β* is a *p*+1 dimensional column vector of unknown parameters to be estimated as *E*(*y*_*i*_)=*μ*.

The variables represented by *X*_*i*_ in equation (2) were *maternal age, region, husband education, mother education, wealth index, media exposure, pregnancy complication, distance from a healthcare facility, plan of pregnancy, and peer influence.*

For *X* is *n* × (*p*+1) matrix of explanatory variables, the link between *y*_*i*_ and *i*^th^ row vector of *X* is *x*_*i*_ linked by ln(*μ*_*i*_) which is given as

(3) $$\begin{array}{c} \ln\left(\mu_{i}\right)=\eta_{i}=\beta_{0}+x_{i}^{T}\beta =\beta_{0}+\beta_{1}x_{1i}+\beta_{2}x_{i1}+\cdots +\beta_{k}x_{ik}, \end{array}$$

where *β*_0_ is the overall intercept, and the *β* is the vector of regression coefficients.

The estimation has taken place by using the maximum likelihood method. Although the Poisson model has served as a place to begin for the frequency analysis for several decades, researchers have often found that count data exhibit characteristics that make the applying of the straightforward Poisson regression problematic. Specifically, the Poisson models cannot handle over- and underdispersion, and they can be adversely affected by low sample means and may produce biased results in small samples. Therefore, it should be performed using some appropriate modeling procedure.

The negative binomial model is an extension of the Poisson model to overcome possible overdispersion in the data [14]. If a Poisson regression model does not fit the data and it appears that the variance of *Y* is increasing faster than the Poisson model allows (i.e., if a plot of the residuals versus linear predictors appears to fan out), then a simple scale-factor adjustment is not appropriate. One way to handle this situation is to fit a parametric model that is more dispersed than the Poisson. A natural choice is the negative binomial [15].

There are situations where a major source of overdispersion is a relatively large number of zero counts, and the resulting overdispersion cannot be modeled accurately with the negative binomial model. In such cases, one can use a zero-inflated Poisson or zero-inflated negative binomial model to fit the data. Zero-inflated distributions can be formed from a component mixture of two distributions. They allow for zero-inflated data and involve a mixture of two distributions where the zeros are modeled separately from the counts.

The zero-inflated Poisson model, well described by Lambert, is a simple mixture model for count data with excess zeros. The model is a combination of a Poisson distribution and a degenerate distribution at zero [16]. Specifically, if *Y*_*i*_ is the number of ANC visits per pregnant mother which are dependent random variables having a zero-inflated Poisson distribution, the zeros are assumed to arise in two ways corresponding to distinct underlying states. The zeros from the first state are called structural zeros and those from the Poisson distribution are called sampling zeros [17].

The zero-inflated negative binomial (ZINB) model is a general model for counts which nests the zero-inflated Poisson (ZIP), negative binomial (NB), and Poisson models. The zero-inflated negative binomial (ZINB) distribution is not a standard generalized linear model (GLM) type, even when the overdispersion parameter *δ* is known, and standard GLM fitting methods are not applied. The method of Fisher scoring is more appropriate for zero-inflated negative binomial regression because the second derivative *l*=(*δ*, *μ*_*i*_, *ω*_*i*_; *y*_*i*_) is simplified by taking expectations [18, 19].

Poisson Logit Hurdle (PLH) model is a two-component model comprising a hurdle component models zero versus nonzero counts, and a truncated Poisson count component is employed for the nonzero counts [20, 21]. Its probability density function is given as

(4) $$\begin{array}{c} p\left(Y_{i}=y_{i}\right)=\left\{\begin{array}{cc} \pi_{0}, & \text{if }y_{i}=0,\\ \left(1-\pi_{0}\right)\frac{\exp\left(-\mu_{i}\right)\mu_{i}^{y_{i}}}{\left(1-\exp\left(-\mu_{i}\right)y_{i}!\right.}\;, & \text{if }y_{i}=1,2,\ldots ,0\leq \pi_{0}\leq 1. \end{array}\right. \end{array}$$

Similarly, for the hurdle model, the hurdle negative binomial can be used instead of the Poisson distribution above in case of overdispersion [20].

**Figure 1:**
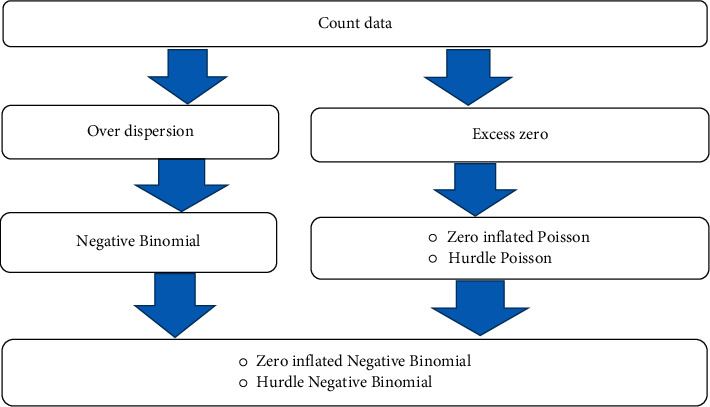
Conceptual framework for count regression models.

The conceptual framework for fitting the count regression model used to show the relationship and difference between different count regression models is shown in Figure 1.

## 3. Results

### 3.1. Prevalence of Antenatal Care Service Visits of Rural Women in Ethiopia

In this study, 5667 women were considered to determine factors associated with barriers in attending the proper number of ANC service visits in rural Ethiopia. This finding revealed that approximately 42% of mothers did not visit any antenatal care service, and 3303(58.28%) women attended one and above the number of ANC service visits. This study result has also shown that 1% of mothers attended eight times and above ANC service visits. In addition, the variance of the dependent variable is greater than the mean, which implies that there is the possibility of overdispersion (Table 1).

Among 1498 women in the age range 15–24, 546 (36.4%) women did not visit any ANC service and only 18 (1.2%) women visited the service eight times and above. The majority of the respondents were in the age range 25–34, and from those mothers in the age group 25–34, 1083 (40.5%) of them did not visit the service. From 1494 mothers in the age group 35–49, only 10 (0.7%) visited the recommended number of the service.

The percentage of women who did not attend ANC service follow-up was 49.3% for those mothers not educated, 25.5% for those with primary education, 16.7% for those with secondary, and 12.2% for those mothers having higher education level.

Concerning region, women living in Afar and Somali had the highest proportion of not attending ANC service visits 352 (61.6%) and 421 (65.2%) women, respectively) while women from Tigray had the lowest proportion of not attending ANC service visits 77 (12%). Additionally, among 175 women from Dire Dawa city administration, 4 (2.3%) had visited the service eight times and above.

Regarding the wealth index, from 3234 poor women, more than half 1660 (51.3) of the respondents did not visit the ANC service while among 1557 richer women, about one-fourth 407 (26.1) of them visited the antenatal care service visits eight times and above (Table 2).

### 3.2. Model Selection Criteria

The adequacies of the models included in this study were compared by using the computed values of Akaki information criteria **(**AIC) and Bayesian information criteria (BIC). To compare the performance of the nonnested model, the researcher used the Voung test [22]. Finally, the hurdle Poisson model is selected by using different model section criteria. Accordingly, the hurdle Poisson model fits the data well to identify the barriers in the number of antenatal care service visits. Procedures for model selection criteria are available in supplementary (available here) files in the form of tables.

**Figure 2:**
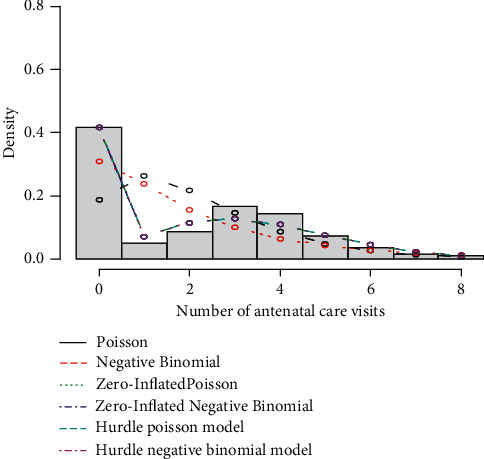
Histogram of the number of ANC visits with overlaid predicted probabilities from each count regression model.

The plots of difference between predicted and observed values from each model against the observed value of the response were used to visualize how the model adequately expresses the response variable. The graph of ZIP, ZINB, HP, and HNB regression models for the differences between predicted and observed values looks overlaid which means that all four regression models efficiently predicted the count of ANC visits per mother. In addition, it is shown that the ZIP, ZINB, HP, and HNB regression models account for the excess zeros quite well and all four regression models reasonably capture the shape of the distribution of the relative frequencies. A hurdle model can account for the excess zeros, and thus, the hurdle Poisson (HIP) might be a solution because it can account for the excess zeros and it provides a more flexible estimator for the variance of the response variable (Figure 2).

### 3.3. Addressing the Existence of Overdispersion using Rootogram

**Figure 3:**
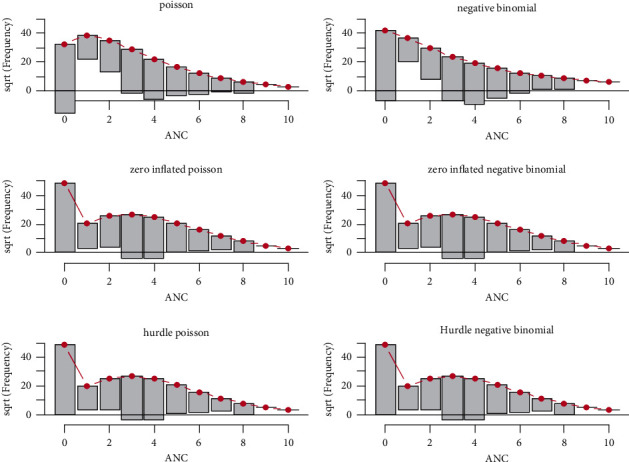
Rootogram to visualize the fit of the hurdle Poisson model.

The rootogram for the hurdle model shows generally good agreement between the expected and observed counts, with a small amount of overprediction of some counts between 1 and 2. The fit of the Poisson GLM to data generated using a ZIP, ZINB, and HNB shows a considerable good fit similar to the hurdle model (Figure 3).

### 3.4. Factors Associated with the Number of Antenatal Care Service Visits in Rural Ethiopia

The predictor variables like wealth index, pregnancy complication, husband education level and age of the mother, educational level of the mother, media access, and distance from a health facility were identified as statistically significant.

The adjusted odds ratio for women who had media access in their surroundings was AOR = 1.745, *P* value = 0.019, which implies that women who had media access were 1.745 times more likely to be experienced to the number of ANC visits compared to women without any media access in their surroundings. There was a significant relationship between the antenatal care service and wealth index. Accordingly, the number of ANC service visits increases as the mother's economy increases. On the other hand, it indicated that the likelihood of antenatal care service visits increases as the educational level of a mother and husband increases. Thus from the results, a woman whose husbands' education level was primary, secondary, and higher increases the number of antenatal care service visits (AOR = 1.018, AOR = 1.117, and AOR = 1.191) compared to husbands with no education, respectively (Table 3)

The adjusted odds ratio for women from the Afar region is AOR = 0.141 (*P* value ≤0.001); Amhara region is AOR = 0.262 (*P* value ≤0.001), Oromia region is AOR = 0.115 (*P* value ≤0.001), Gambela region is AOR = 0.106 (*P* value ≤0.001), Harari region is AOR = 0.194 (*P* value ≤0.001), Somali region is AOR = 0.1081 (*P* value ≤0.001), and Benshangul is AOR = 0.256 (*P* value ≤0.001) (SNNPR = 0.244, *P* value ≤0.001). This implies that most women in the rural area of Ethiopia did not visit antenatal care services appropriately compared to Tigray region women (Table 3).

The likelihood of the number of antenatal care service visits for mothers in the age group 35–49 was 0.690 times lower as compared to the number of antenatal care service visits for mothers in age groups 15–19 (AOR = 0.690, *P* value ≤0.001). The rate ratio for childbearing mothers having pregnancy complications during pregnancy is AOR = 3.114 (*P* value ≤0.001), which implies that those mothers having pregnancy complications were 3.114 times more likely to visit ANC service than those mothers who have no pregnancy complication. The adjusted odds ratio for fertile aged mothers having distance problems is AOR = 0.75 (*P* value = 0.013), which implies that those mothers having distance problems were 0.75 times less likely to visit ANC service than those mothers who have no distance problems (Table 3).

According to the results, the number of ANC follow-ups was higher for mothers with an educational level of primary and above compared with mothers with no education. The adjusted odds ratio for mothers having primary education is AOR = 1.85 (*P* value ≤0.001) and that for those mothers having secondary education level is AOR = 2.387 (*P* value ≤0.001). This implies that women who attended primary and secondary formal education were 1.85 and 2.387 times more likely to visit ANC service compared to women who have not attended education, respectively (Table 3).

## 4. Discussion

Using the EDHS data and an appropriate modeling approach, this study further assessed factors affecting mothers in attending a recommended number of ANC services visits in Ethiopia. Thus, this study attempted to identify sociodemographic, fertility-related characteristics, and ANC service-related determinants of completing the recommended number of ANC service visits among pregnant women of reproductive age in rural parts of Ethiopia.

This study indicated that the number of ANC services is strongly influenced by the mother's history of pregnancy complications. Mothers who had a history of pregnancy complications most probably attended the number of ANC service visits properly (AOR = 3.114, *P* value ≤0.001). This result agreed with the previous studies that were undertaken in Ecuador and Taiwan [23, 24]. This is probably because those women who experienced pregnancy complications were more concerned about their health and better perceived the risk of pregnancy. As a result, they are more probably keen to seek medical care early and regularly.

Media access had a positive influence on women to utilize ANC service (AOR = 1.745, *P*=0.0185). Already conducted findings in Ethiopia and Nigeria have seen the impact of media introduction on ANC visits [25, 26]. The visit advancement of maternal well-being administrations through media might impact women's inclination for an early visit and their adherence to consequent follow-ups by giving them important data.

Women's educational level was one of the strong predictors of attending ANC visit services follow-up in the study. The number of ANC service visits was higher for mothers with the educational level of primary (AOR = 1.85, *P* value ≤0.001) and secondary (AOR = 2.387, *P* value ≤ 0.001) compared with uneducated mothers. This finding was consistent with a previous study done in Central Ethiopia which found that women with some education were more than twice more likely to attend ANC (OR = 2.645) as compared with those who had no education [25]. The study conducted in Nepal demonstrated that women with higher education were more likely to utilize ANC than those with lower education [27]. A study carried out by Pallikadavath et al. [28] found similar results; in their study, they had demonstrated that both maternal and paternal education positively influence utilization of ANC service visits. Another factor for attending ANC service visits in the country was the wealth index. The wealth index was strongly and negatively associated with the utilization of ANC services in rural Ethiopia. This study showed that women in middle and richer economic status were more likely to attend ANC service than those of poorer women. It is in line with several studies in different countries [25]. Similarly, a study from China found that women who had higher household incomes were more likely to have sufficiently utilized ANC services (AOR = 1.631, CI = 1.0–2.5) [29].

Distance from health facility was an important sociodemographic predictor of barriers in the number of antenatal care service visits; that is, mothers having distance problems were 0.75 times less likely to attend ANC service visits compared with those mothers who had no distance problem (AOR = 0.75, CI = 0.73,0.98). This result is in line with the previous study, which indicated that the distance from the health facility is inversely associated with ANC utilization [30], and also finding conducted in Magadi in Kenya [31] which demonstrated that an increase in distance to the nearest healthcare facilities was associated with fewer antenatal visits.

Regarding this study, older women were less likely to have adequately utilized antenatal care rather than the younger. The estimated odds of the number of antenatal care service visits among mothers in the age group 35–49 are 0.690 times lower than that of mothers in the age group 15–19. This result is contradicting several previous studies. A study from Central Ethiopia found that the odds of attending ANC are 1.2 times higher (OR = 1.168) for women in the age group of 20–34 as compared to those in the age group 15–19 women [25]. Likewise, a study conducted in Vietnam found that older women (more than 25 years old) were more likely to utilize antenatal care [9]. This may be due to the adaptation of pregnancy time variations and the distance from the health facility since the study was conducted in the rural parts. The reason might be the fact that older mothers have births experience and they may not give concentration for the necessities of the service.

## 5. Conclusion

Out of the total participants included in the study, only 59 (1%) women attended ANC services eight times and more. Approximately, 42% of mothers did not attend ANC services and 58.3% visited only once. This figure showed the underutilization of ANC services in rural parts of the country as compared to the targeted number of ANC services visits.

The hurdle Poisson model was selected to account for the overdispersion and excess zero of the number of antenatal care service visits in rural Ethiopia. Based on the hurdle Poisson regression results, the covariates like age, wealth index, mother education, husband education, media access, distance from the health facility, plan of pregnancy, region, and pregnancy complication were found to be related to the antenatal care service follow-up. Our study indicated that there are barriers in terms of antenatal care service visits in rural parts of Ethiopia. Expansion of infrastructure among the rural community needs to be prioritized by the concerned body and the government of Ethiopia has to expand the media coverage related to ANC throughout the country and mothers must bear in mind the importance of ANC during pregnancy.

## 6. Weaknesses and Strengths of this Study

One of the potential weaknesses of this study is the cross-sectional nature of our analysis. The study uses reported characteristics of mothers that may vary over time. The study used data from national surveys that have inherent gaps such as the absence of some variables that may affect the response variable and some variables are not included because of the large number of missing values like parity. The major strengths of this study were the availability of pieces of kinds of literature and it is the current issue to visit antenatal care services eight times and above.

## Figures and Tables

**Table 1:** Frequency distribution of the number of antenatal care visits per woman.

Number of ANC service visits	Number of mothers that are experienced with ANC	Percent
0	2364	41.7
1	281	5.0
2	484	8.5
3	949	16.7
4	820	14.5
5	415	7.3
6	205	3.6
7	90	1.6
8+	59	1.0
Total	5667	100
Mean = 2.08	Variance = 4.49	

**Table 2:** Summary statistics of the number of antenatal care visits and associated risk factors among rural women in count and percent.

		Number of antenatal care visits									Total
		0	1	2	3	4	5	6	7	8+	
		Count (%)	Count (%)	Count (%)	Count (%)	Count (%)	Count (%)	Count (%)	Count (%)	Count(%)	
Maternal age	15–24	546(36.4)	88(5.9)	140(9.3)	280(18.7)	234(15.6)	121(8.1)	49(3.3)	22(1.5)	18(1.2)	**1498**
	25–34	1083(40.5)	131(4.9)	230(8.6)	456(17.0)	392(14.7)	192(7.2)	110(4.1)	50(1.9)	31(1.2)	**2675**
	35–49	735(49.2)	62(4.1)	114(7.6)	213(14.3)	194(13.0)	102(6.8)	463(3.1)	18(1.2)	10(0.7)	**1494**

Mother's education level	No education	1959(49.3)	211(5.3)	338(8.5)	563(14.2)	464(11.7)	230(5.8)	119(3.0)	56(1.4)	34(0.9)	**3974**
	Primary	362(25.5)	61(4.3)	129(9.1)	321(22.6)	282(19.8)	148(10.4)	74(5.2)	28(2.0)	17(1.2)	**1422**
	Secondary	37(16.7)	5(2.3)	15(6.8)	52(23.4)	63(28.4)	28(12.6)	10(4.5)	5(2.3)	7(3.2)	**222**
	Higher	6(12.2)	4(8.2)	2(4.1)	13(26.5)	11(22.4)	9(18.4)	2(4.1)	1(2.0)	1(2.0)	**49**

Pregnancy complications	No	1983(50.5)	224(5.7)	350(8.9)	558(14.2)	432(11.0)	205(5.2)	109(2.8)	42(1.1)	25(0.6)	**3928**
	Yes	381(21.9)	57(3.3)	134(7.7)	391(22.5)	388(22.3)	210(12.1)	96(5.5)	48(2.8)	34(2.0)	**1739**

Husband/partner's education level	No education	1598(52.2)	163(5.3)	259(8.5)	406(13.3)	327(10.7)	159(5.2)	96(3.1)	35(1.1)	20(0.7)	**3063**
	Primary	605(32.2)	98(5.2)	179(9.5)	384(20.4)	326(17.4)	161(8.6)	66(3.5)	38(2.0)	21(1.1)	**1878**
	Secondary	105(22.6)	13(2.8)	31(6.7)	106(22.8)	116(25.0)	50(10.8)	23(5.0)	10(2.2)	10(2.2)	**464**
	Higher	56(21.4)	7(2.7)	15(5.7)	53(20.2)	51(19.5)	45(17.2)	20(7.6)	7(2.7)	8(3.1)	**262**

Region	Tigray	77(12.2)	41(6.5)	67(10.6)	131(20.8)	160(25.4)	95(15.1)	42(6.7)	9(1.4)	9(1.4)	**631**
	Afar	352(61.6)	44(7.7)	51(8.9)	56(9.8)	30(5.3)	22(3.9)	9(1.6)	2(0.4)	5(0.9)	**571**
	Amhara	254(36.9)	36(5.2)	60(8.7)	152(22.1)	81(11.8)	53(7.7)	23(3.3)	19(2.8)	10(1.5)	**688**
	Oromia	494(51.0)	42(4.3)	71(7.3)	161(16.6)	105(10.8)	57(5.9)	29(3.0)	5(0.5)	4(0.4)	**968**
	Somali	421(65.2)	43(6.7)	55(8.5)	74(11.5)	27(4.2)	14(2.2)	7(1.1)	2(0.3)	3(0.5)	**646**
	Benshangul	184(34.3)	16(3.0)	34(6.3)	101(18.8)	137(25.6)	49(9.1)	8(1.5)	5(0.9)	2(0.4)	**536**
	SNNPR	254(31.2)	25(3.1)	79(9.7)	148(18.2)	177(21.8)	68(8.4)	36(4.4)	17(2.1)	9(1.1)	**813**
	Gambela	199(50.6)	9(2.3)	25(6.4)	57(14.5)	52(13.2)	24(6.1)	19(4.8)	5(1.3)	3(0.8)	**393**
	Hareri	88(35.8)	21(8.5)	33(13.4)	44(17.9)	21(8.5)	12(4.9)	7(2.8)	10(4.1)	10(4.1)	**246**
	Dire dawa	41(23.4)	4(2.3)	9(5.1)	25(14.3)	30(17.1)	21(12.0)	25(14.3)	16(9.10	4(2.3)	**175**

Media exposure	Not exposed	2336(42.5)	272(5.0)	472(8.6)	906(16.5)	783(14.3)	391(7.1)	196(3.6)	82(1.5)	54(1.0)	**5492**
	Exposed	28(16.0)	9(5.1)	12(6.9)	43(24.6)	37(21.1)	24(13.7)	9(5.1)	8(4.6)	5(2.9)	**175**

Distance to health facility	No problem	272(44.2)	38(6.2)	43(7.0)	104(16.9)	91(14.8)	36(5.8)	23(3.7)	4(0.6)	5(0.8)	**616**
	Problem	2092(41.4)	243(4.8)	441(8.7)	845(16.7)	729(14.4)	379(7.5)	182(3.6)	86(1.7)	54(1.1)	**5051**

Peer influence	No problem	13(27.7)	4(8.50)	3(6.4)	5(10.6)	12(25.5)	7(14.9)	2(4.3)	1(2.1)	0(0.0)	**47**
	Problem	2351(41.8)	277(4.9)	481(8.6)	944(16.8)	808(14.4)	408(7.3)	203(3.6)	89(1.6)	59(1.0)	**5620**

Wealth index	Poor	1660(51.3)	186(5.8)	275(8.5)	453(14.0)	348(10.8)	166(5.1)	87(2.7)	34(1.1)	25(0.8)	**3234**
	Middle	297(33.9)	33(3.8)	82(9.4)	167(19.1)	158(18.0)	66(7.5)	44(5.0)	20(2.3)	9(1.0)	**876**
	Richer	407(26.1)	62(4.0)	127(8.2)	329(21.1)	314(20.2)	183(11.8)	74(4.8)	36(2.3)	25(1.6)	**1557**

Plan of the pregnancy	No	224(48.7)	20(4.3)	27(5.9)	67(14.6)	72(15.7)	23(5.0)	16(3.5)	9(2.0)	2(0.4)	**460**
	Yes	2140(41.1)	261(5.0)	457(8.8)	882(16.9)	748(14.4)	392(7.5)	189(3.6)	81	57(1.1)	**5207**

**Table 3:** Factors associated with the number of ANC service visits (0,1,2,…, 8+) in rural parts of Ethiopia.

Count regression result (Hurdle–Poisson regression model)				
Variables	Categories	Estimates	AOR (95% CI for OR)	*P* value
Pregnancy complication	Intercept	1.140	3.128 (2.952, 3.315)	≤0.001^*∗*^
	No	Ref	1	
	Yes	0.145	1.156(1.111, 1.203)	≤0.001^*∗*^

Husband education	No education	Ref	1	
	Primary	0.018	1.018(1.035, 1.864)	0.004^*∗*^
	Secondary	0.110	1.117(1.047, 1.191)	≤0.001^*∗*^
	Higher	0.175	1.191(1.102, 1.287)	≤0.001^*∗*^

Wealth index	Poorer	Ref	1	
	Middle	0.077	1.080(1.022, 1.142)	0.006^*∗*^
	Richer	0.095	1.100(1.050, 1.152)	≤0.001^*∗*^

Region	Tigray	Ref	1	
	Afar	−0.176	0.839(0.760, 0.926)	≤0.001^*∗*^
	Amhara	−0.045	0.956(0.890, 1.026)	0.211
	Oromia	−	0.906(0.845, 0.972)	0.006^*∗*^
	Somali	−0.283	0.753(0.682, 0.832)	≤0.001^*∗*^
	Benishangul	−0.035	0.965(0.897, 1.039)	0.349
	SNNPR	−0.021	0.979(0.918, 1.045)	0.531
	Gambela	0.003	1.00(0.917, 1.099)	0.940
	Harari	−0.092	0.912(0.825, 1.008)	0.073
	Dire dawa	0.280	1.323(1.205, 1.453)	≤0.001^*∗*^

Logistic regression result				
Maternal age	Intercept	2.000	7.392(3.386, 16.140)	≤0.0001^*∗*^
	15–24	Ref	1	
	25–34	0.0157	1.016(0.870, 1.187)	0.843
	35–49	−0.37051	0.690(0.577, 0.827)	≤0.001^*∗*^

Mothers education	No education	Ref	1	
	Primary	0.617	1.85(1.574, 2.183)	≤0.001^*∗*^
	Secondary	0.8701	2.387(1.560, 3.654)	≤0.001^*∗*^
	Higher	0.780	2.182(0.815, 5.84)	0.120

Media exposure	Not exposed	*Ref*	1	
	Exposed	0.556	1.745(1.098, 2.775)	0.019^*∗*^

Distance from health facility	No problem	Ref	1	
	Problem	−0.289	0.75(0.73,0.98)	0.013^*∗*^

Pregnancy complication	No	Ref	1	
	Yes	1.136	3.114(2.703, 3.587)	≤0.001^*∗*^

Husband education	No education	Ref	1	
	Primary	0.426	1.531(1.327, 1.767)	≤0.001^*∗*^
	Secondary	0.861	2.366(1.811,3.092)	≤0.001^*∗*^
	Higher	0.807	2.244(1.553, 3.236)	≤0.001^*∗*^

Plan of pregnancy	Not planned	Ref	1	
	Planned	0.353	1.42(1.144, 1.770)	0.002^*∗*^

Wealth index	Poorer	Ref	1	
	Middle	0.489	1.631(1.360, 1.957)	≤0.001^*∗*^
	Richer	0.739	2.094(1.777, 2.467)	≤0.001^*∗*^

Region	Tigray	Ref	1	
	Afar	−1.959	0.141(0.103, 0.193)	≤0.001^*∗*^
	Amhara	−1.341	0.262(0.193, 0.354)	≤0.001^*∗*^
	Oromia	−2.158	0.115(0.086, 0.154)	≤0.001^*∗*^
	Somali	−2.225	0.1081(0.080, 0.147)	≤0.001^*∗*^
	Benishangul	−1.364	0.256(0.186, 0.351)	≤0.001^*∗*^
	SNNPR	−1.410	0.244(0.181, 0.330)	≤0.001^*∗*^
	Gambela	−2.240	0.106(0.076, 0.150)	≤0.001^*∗*^
	Harari	−1.638	0.194(0.132, 0.2853)	≤0.001^*∗*^
	Dire dawa	−0.438	0.645(0.414, 1.004)	0.052

Ref: reference categories, AOR: adjusted odds ratio, CI: confidence interval, *∗*significance at 5%.

## Data Availability

The datasets used in this study are available from the corresponding author on reasonable request.

## Abbreviations

AIC: Akaki information criteria
ANC: Antenatal care
BIC: Bayesian information criteria
EDHS: Ethiopian demographic and health survey
HP: Hurdle–Poisson
HNB: Hurdle negative binomial
WHO: World Health Organization
ZIP: Zero-inflated Poisson
ZINB: Zero-inflated negative binomial.

## Supplementary Materials

Supplementary Materials The adequacies of the models included in this study were compared by using the computed values of Akaki information criteria **(**AIC) and Bayesian information criteria (BIC). The model with the smallest AIC and BIC was considered as the best model to fit the dataset. Therefore, the hurdle Poisson regression model was identified as a good model to fit the dataset. To compare the performance of the nonnested model, the researcher used the Voung test. After a series of tests and model comparisons, the HP model is preferred to the ZIP regression model (*Z* = −0.613, *P*=0.027). Thus, the HP model was selected as a good model and is strengthened by AIC and BIC values. The Poisson and NB model underestimated zero counts, the zero-inflated models overestimated zero counts, and the hurdle models captured all zero values. Based on predicted outcomes, the differences in model fit between the six models are remarkable. Still, the standard Poisson model and the NB model do not fit the data reasonably well.
