# Supplementary material for: Identifying Factors Associated with Barriers in the Number of Antenatal Care Service Visits among Pregnant Women in Rural Parts of Ethiopia
Source: ScientificWorldJournal. 2021 Oct 25;2021:7146452. doi: 10.1155/2021/7146452 (PMC8560300; doi:10.1155/2021/7146452)
Supplement: Supplementary Materials — The adequacies of the models included in this study were compared by using the computed values of Akaki information criteria (AIC) and Bayesian information criteria (BIC). The model with the smallest AIC and BIC was considered as the best model to fit the dataset. Therefore, the hurdle Poisson regression model was identified as a good model to fit the dataset. To compare the performance of the nonnested model, the researcher used the Voung test. After a series of tests and model comparisons, the HP model is preferred to the ZIP regression model (Z = −0.613, P=0.027). Thus, the HP model was selected as a good model and is strengthened by AIC and BIC values. The Poisson and NB model underestimated zero counts, the zero-inflated models overestimated zero counts, and the hurdle models captured all zero values. Based on predicted outcomes, the differences in model fit between the six models are remarkable. Still, the standard Poisson model and the NB model do not fit the data reasonably well. [file 7146452.f1.docx]

**Model selection criteria**

The adequacies of the models included in this study were compared by using the computed values of Akaki Information Criteria **(**AIC) and Bayesian information criteria (BIC). To compare the performance of the non-nested model, the researcher used the Voung test. The model with the smallest AIC and BIC was considered as the best model to fit the dataset. Therefore, the Hurdle Poisson regression model was identified as a good model to fit the dataset

**Table :** The AIC and BIC values for model comparison

| Model | AIC | BIC |
| --- | --- | --- |
| Poisson | 22094.64 | 22267.35 |
| NB | 20891.52 | 21070.86 |
| ZIP | 18248.32 | 18587.92 |
| ZINB | 18250.32 | 18602.37 |
| **HP** | **18242.51** | **18558.88** |
| HNB | 18244.51 | 18596.56 |

**Model performance checking**

To compare the performance of the non-nested model, the researcher used the Voung test. After a series of tests and model comparisons, the HP model is preferred to the ZIP regression model (Z= -0.613, P=0.027). Thus, the HP model was selected as a good model and is strengthened by AIC and BIC values**.**

**Table:** Voung test of the non-nested models

| Model | Vuong test statistic | p-value | Preferable model |
| --- | --- | --- | --- |
| Poisson vs ZIP  NB VS ZINB  ZINB VS HNB  ZIP VS HP | -34.217  -40.290  -3.028  -0.613 | ≤0.001  ≤0.001  ≤0.001  0.027 | ZIP  ZINB  HNB  HP |

**Observed and predicted values of the data**

As shown in the Poisson and NB model under-estimated zero counts, the zero-inflated models over-estimated zero counts and the hurdle models captured all zero values. Based on predicted outcomes, the differences in model fit between the six models are remarkable. Still, the standard Poisson model and the NB model do not fit the data reasonably well.

**Table:** Zero counts capturing in count models.

| Number of zeros | Observed | Poisson | NB | ZIP | NB | HP | HNP |
| --- | --- | --- | --- | --- | --- | --- | --- |
|  | 2364 | 1067.376 | 1753.681 | 2366.709 | 2366.688 | 2364 | 2364 |
